# Prevalence of overweight and obesity amongst patients with diabetes and their non-diabetic family members in Senwabarwana, Limpopo province, South Africa

**DOI:** 10.4102/safp.v64i1.5409

**Published:** 2022-05-25

**Authors:** Mabitsela H. Mphasha, Linda Skaal, Tebogo M. Mothiba

**Affiliations:** 1 Department of Public Health, Faculty of Healthcare Sciences, University of Limpopo, Polokwane, South Africa; 2 Faculty of Healthcare Sciences, University of Limpopo, Polokwane, South Africa

**Keywords:** patients with diabetes, overweight, obesity, family members, body mass index

## Abstract

**Background:**

Diabetes remains a public health concern and the second cause of mortality in South Africa. Family history of diabetes increases risk of developing diabetes. Obesity amongst patients is associated with comorbidity, whilst amongst non-diabetic family members it is associated with developing diabetes. This study aimed at determining prevalence of overweight and obesity amongst patients with diabetes and non-diabetic family members.

**Methods:**

A quantitative, cross-sectional descriptive study was conducted on 200 patients and 200 non-diabetic family members were selected using systematic random sampling from rural clinics of Senwabarwana. Data were collected using close-ended questionnaires and anthropometric measurements. Body mass index (BMI) and waist circumference were measured and interpreted according to World Health Organization guidelines. Data were analysed using Statistical Package for Social Sciences, using both descriptive and inferential statistics. Chi-square test was used to calculate associations at 95% confidence interval where a *p*-value of < 0.05 was considered statistically significant.

**Results:**

Most patients (75.5%) had comorbidities and hypertension was most prevalent (89.0%). Over half of the patients (57.0%) and 38.0% of family members were obese. Most patients (75.0%) and 58.0% of family members had abdominal obesity.

**Conclusion:**

Patients with diabetes suffer from comorbidities are overweight and obese whilst evidence from various studies suggest that non-diabetic family members are at added risk of developing diabetes because of higher BMI and abdominal obesity. There is an urgent need to create a conducive environment that discourages sedentary behaviours through lifestyle modifications using the family centred approach, and involve family members in the care of patients.

## Introduction

The International Diabetes Federation^1^ posits that rising diabetes mellitus (DM) prevalence remain a public health concern and the second cause of mortality in South Africa (SA).^2^ In 2019, Africa had approximately 19 million adults aged 20–79 years personally diagnosed or living with diabetes. South Africa has the highest number of patients with type-2 diabetes mellitus (T2DM) (estimated at 4.6 million),^1^ but only 2 million people have been diagnosed.^3,4^ Africa has the greatest proportion of undiagnosed diabetics and projections suggest it will experience the greatest future increase in the burden of DM of 156% by 2045.^5^

Obesity is central to DM prevalence and one of the main predisposing factors for all non-communicable diseases (NCDs).^6^ Obesity has emerged as a public health concern with global prevalence of 2 billion adults being overweight, and of those, 650 million are considered to be obese (body mass index [BMI] ≥ 30 kg/m²).^7^ In Africa, SA is amongst the countries with the highest obesity prevalence.^8^ A South African study has reported that 56%, 49% and 75% of white men, African men and women were overweight or obese, respectively.^9^ Obesity prevalence amongst Africans was attributed to cultural attitudes towards fatness.^8^ It was found that culture affects body image and size perceptions from an early age and that exclusive body shape and weight ideals are recognised amongst different cultures.^10^ African women regard overweight as being rich, healthy, of good strength and fertility,^11^ including being beautiful and attractive^8^ and also of normal weight.^12^ Therefore, these could be the fuelling factors for increasing obesity and overweight levels in Africa. The increase of disposable income resulting in more money being spent on high energy-dense foods is the key factor in the rise in obesity in SA, as in the rest of the world. Another South African study reported a higher prevalence of obesity amongst urban women, compared with rural women^13^ and attributed these to reduced intake of fatty food, lower household income and higher physical activity.^13^ Obesity may additionally result from poor eating habits and physical inactivity.^14^ It has been reported that relentless obesity upsets metabolic processes that control blood glucose, blood pressure and lipids.^15,16^

The fact that the incidence of diabetes is rising so rapidly with changes in eating habits and lifestyle suggests that genetic and hereditary elements are of much less importance than diet and behaviour changes. It was determined that obesity and diabetes have strong environmental components, inclusive of diet and physical activity. Family members share similar genetical backgrounds and often similar environmental and lifestyle factors affecting their health. Therefore, family history of diabetes and occurrence of obesity may guide population-appropriate health promoting activities.^17,18,19^ The environmental risk factors associated with diabetes include obesity, sedentary lifestyle, small or large birth weight and stress.^20^ However, these environmental factors impact people differently; some people are more susceptible to diabetes and risks are inherited.^21^ The lifetime risk of developing diabetes is 70% amongst individuals with father and mother diagnosed with diabetes compared with 40% of those with one parent.^22^ Non-diabetic family members of patients are predisposed because of the existing family history of DM.^22^ It is far too easy to blame genetics, when the problem is really the lifestyle behaviours – a diet deficient in fibre, ultra-processed foods, high in saturated fats and animal proteins, together with lack of physical activity. It is important to address these, even if it is not ‘politically expedient’, as this kind of diet is seen as a sign of social upward mobility. A healthy eating and increased physical activity can counteract these genetic effects.^17^ Individuals with a family history of diabetes are three times more likely to develop diabetes than those without a family history.^23^ Rural areas such as Senwabarwana in the Limpopo province of SA, have been progressively urbanised,^24^ which has led to the adoption of unhealthy lifestyles resulting in high incidences of obesity that contributes to poor diabetes outcomes and complications.^6,25^ It was found that higher BMI amongst diabetes patients is associated with diabetic complications such as cardiovascular diseases.^26^ This article aimed at determining prevalence of overweight and obesity amongst patients with diabetes and their non-diabetic family members.

## Methods

### Research design

A quantitative approach and cross-sectional study descriptive design were used.

### Study participants and setting

A total of 400 participants (200 diabetics and 200 non-diabetic family members) were included in this study. Diabetes patients were sampled from a population of 406 receiving treatment at the clinics, using a systematic random sampling technique where every second patient was recruited verbally. The selected patients with diabetes were requested to bring their non-diabetic family members who are not on treatment and above the age of 18 years. The family members included children and siblings of patients with diabetes. The sample size of patients was calculated using Taro Yamane formula^27^ at 95% confidence interval (CI) formula is as follows:

$$n=N÷1+N{\left(e\right)}^{2},$$ [Eqn 1]

where

*n* = sample size

*N* = population size (*N* = 406)

*e* = error margin (5%)

The sample size for patients was 200 and additional 20 patients were also included in the study to cater for attrition or incomplete questionnaires, which finally yielded 200 participants who were used in the study. The additional sampling for attrition was used to replace the incomplete or spoilt questionnaires. In the study, we included only patients who had lived with DM for six or more months and were aged 18 years and above.

This study was conducted in clinics of Blouberg municipality in Senwabarwana of the Capricorn District and located in the Limpopo province of SA. Senwabarwana is a rural area within Blouberg Municipality, which has a population of 162 297.^28^ Blouberg Municipality is demarcated into 22 municipal wards and has a total of 22 clinics, two health centres, four mobile clinics and one hospital. The clinics are divided into four areas. However, there are municipal wards with more than two clinics including health centres whilst other wards have no clinics. All the clinics refer their patients to the only existing hospital in the municipality.

### Instruments

Data had been gathered using two close-ended questionnaires and anthropometric measurements where BMI and waist circumference (WC) were measured. The first questionnaire was for patients with diabetes whilst the second questionnaire was for family members. All questionnaires had two sections, namely demographic profile and anthropometric measurements. Anthropometric measurements included weight, height and WC; measured using a calibrated electronic weight scale and a tape measure. The calculation of BMI was as follows: BMI = weight (kg)/height (m^2^). The weighing scales were calibrated and measurements for weight were taken thrice using the same brand of electronic weight scale to ensure reliability. A measuring tape was used to measure height and WC. Furthermore, reliability was tested through piloting the questionnaire in non-participating clinics and it yielded no changes. Content and face validity had been ensured through the use of dieticians and supervisors for relevance. Also, content validity was ensured through the literature review.

### Data analysis

Data were coded and entered into the Statistical Package for Social Sciences version 25.0 for analysis. The BMI was classified according to World Health Organization (WHO)^29^ as follows: *Underweight* (< 18 kg/m^2^), *normal weight* (18.5 kg/m^2^ – 24.9 kg/m^2^), *overweight* (25.0 kg/m^2^ – 29.9 kg/m^2^) and *obese* (≥ 30 kg/m^2^).

Also, WC for *abdominal obesity* was classified as per WHO^30^ guidelines: *Male* (*> 102 cm*) and *females* (*> 88 cm*). Descriptive statistics were used where frequency distributions, means and standard deviations were calculated. Chi-squared test was used to calculate associations at 95% CI. A *p*-value of < 0.05 was considered statistically significant.

### Ethical considerations

This study is part of bigger study approved by by Turfloop Research Ethics Committee (TREC) of the University of Limpopo (reference number: TREC/35/2019:PG) and permission was given by Limpopo Department of Health (reference: LP 201903-007). All participants provided written informed consent. Participation was voluntary and participants were informed about their rights to withdraw from the study at any stage without penalty. Privacy and confidentiality of the participant’s data were also maintained.

## Results

### *Demographic profile of* respondents

In Table 1 shows that most of the patients (65.0%), and 27.0% of family members were of the age ≥ 61 years. Over half of patients (53.0%) and 34.0% of family had primary education. Majority of patients (74.5%) and 46.0% of family members were married whilst 75.5% of patients with diabetes had comorbidity and hypertension was most prevalent (89.0%).

**TABLE 1:** Demographic profile of respondents.

Demographic data	Patients (*n* = 200)		Family members (*n* = 200)	
	*n*	%	*n*	%
**Age groups (years)**				
≤ 60	70	35.0	146	73
> 61	130	65.0	54	27
**Gender**				
Male	37	18.5	48	24
Female	163	81.5	152	76
**Education**				
No education	57	29.0	04	2
Primary education	105	53.0	68	34
Secondary or higher	38	19.0	128	64
**Marital status**				
Single	51	25.5	108	54
Married	149	74.5	92	46
**Any other chronic illness except diabetes**				
Yes	151	75.5	-	-
No	49	24.5	-	-
**Types of other diseases (*n* = 151)**				
Hypertension	135	89.0	-	-
Arthritis	12	8.0	-	-
Heart	4	3.0	-	-

**FIGURE 1:**
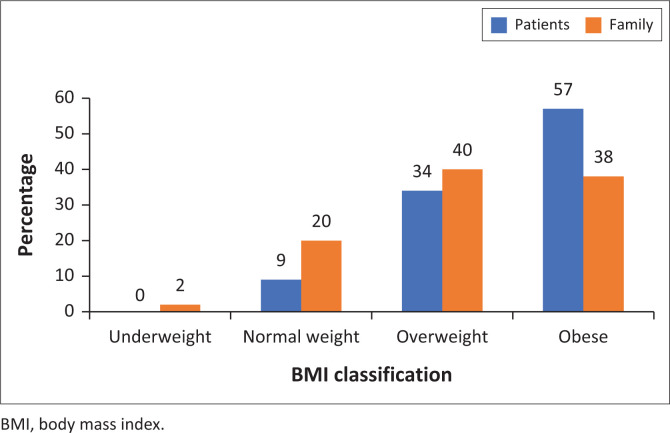
Body mass index of respondents.

Figure 1 shows that 57% of patients with diabetes and 38% of family members were obese. Furthermore, 34% of patients and 40% of family members were overweight.

Table 2 shows significant association of BMI of both patients and family members against their sociodemographic profile. There was no significant association between BMI and age of patients (*p* = 0.171) whilst there was significant association between BMI and age of famißly members (*p = 0.023*). Also, there was a significant association between BMI and marital status of patients and that of family members (*p* = 0.41 and 0.000), respectively.

**TABLE 2:** Body mass index by sociodemographic profile of respondents.

Sociodemographic profile	Patients		Family members	
	*p*	*χ* ^2^	*p*	*χ* ^2^
**Age (years)**				
≤ 30	0.171	3.527	0.023*	19.307
31–50	-	-	-	-
51–70	-	-	-	-
> 70	-	-	-	-
**Gender**				
Male	0.010*	9.300	0.521	2.258
Female	-	-	-	-
**Education**				
No education	0.297	2.427	0.544	4.999
Primary education	-	-	-	-
Secondary or higher	-	-	-	-
**Marital status**				
Single	0.041*	6.376	0.000*	30.376
Marriage	-	-	-	-

* , Signifies statistical significance at 95% confidence interval.

**FIGURE 2:**
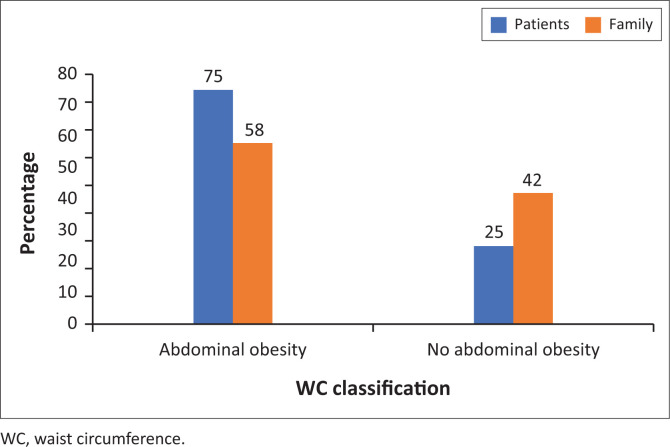
Waist circumference of participants.

Figure 2 shows that 75% of patients with diabetes and 58% of family members had abdominal obesity.

**FIGURE 3:**
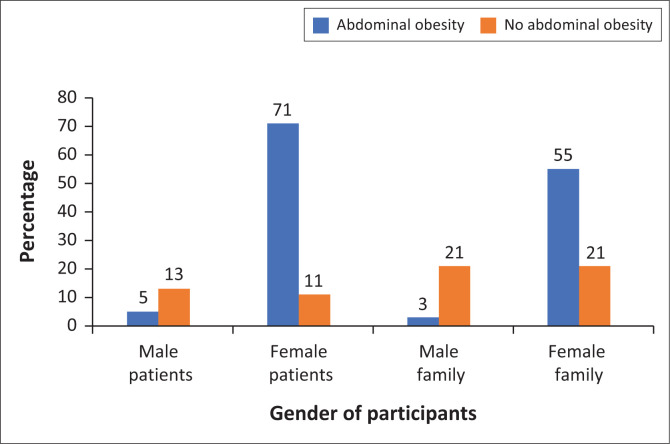
Waist circumference of participants by gender.

Figure 3 shows that most female patients 71% and 55% of female family members had abdominal obesity.

Table 3 shows significant association of WC of both patients and family members against their sociodemographic profile. There was no significant association between WC and age, gender and marital status of patients and family members: *p* = 0.309 and *p* = 0.166, *p* = 0.737 and *p* = 0.656 and *p* = 0.723 and *p* = 0.389, respectively. There was a significant association between WC and the education of patients (*p* = 0.031) whilst there was no significant association between WC and the education of family members (*p* = 3.76).

**TABLE 3:** Waist circumference by sociodemographic profile of respondents.

Sociodemographic profile	Patients		Family members	
	*p*	*χ* ^2^	*p*	*χ* ^2^
**Age (years)**				
≤ 30	0.309	3.592	0.166	5.081
31–50	-	-	-	-
51–70	-	-	-	-
> 70	-	-	-	-
**Gender**				
Male	0.737	0.113	0.656	0.199
Female	-	-	-	-
**Education**				
Primary or less	0.031*	6.921	0.376	1.955
Secondary or more	-	-	-	-
**Marital status**				
Single	0.723	0.125	0.389	0.741
Marriage	-	-	-	-

* , Signifies statistical significance at 95% confidence interval.

## Discussion

A United States prospective cohort study revealed that higher BMI values are associated with diabetes complications and risks of T2DM.^31^ This study is similar to the 2016 WHO^7^ report, which indicated that 39% of adults with or without diseases were overweight because 34% and 40% of patients and family members in our study were found to be overweight. However, in contrast our findings surpass the estimated 13% obesity rates because 57% of patients with diabetes and 38% of family members were obese. Our findings are similar to the Ugandan retrospective study, which reported a high prevalence of overweight and obesity amongst T2DM patients.^32^ It has been reported that overweight and obesity amongst patients with diabetes increase burden of disease and poor outcomes.^33^ Five longitudinal cohort studies conducted have pointed out that overweight or obese patients with diabetes are at a twofold higher risk of mortality compared with those with normal BMI.^33,34^ In addition, overweight and obesity amongst patients with diabetes increase the likelihoods of suboptimal glycaemic control.^33,35^ Furthermore, it has been reported that obesity contributes to insulin resistance and metabolic syndrome whose other components besides hyperglycaemic include hypertension, dyslipidaemia, proinflammatory and prothrombotic state.^35^ Higher BMI amongst both patients and family members can be attributed to sedentary lifestyle and unhealthy eating habits. Lack of commitment to addressing weight management contributes significantly to rising obesity levels. Healthcare providers need to commit to this task. Without urgent intervention, such as dietary and exercise modification, overweight patients may become obese and subsequently increase their risk for diabetes complications and increased mortality.^2,36^ Urgent dietary and exercise modifications include application of 5-A’s methods of effective counselling and behaviour change, which are: (1) assess health behaviours (understand in depth what is going on); (2) advise to change with clear, specific, personalised advice; (3) agree on focus of treatment based on patient’s priorities; (4) assist patient in setting and achieving goals; and (5) arrange regular follow-up and support. This is patient-centred, collaborative approach and thus it is associated with greater patient motivation to change behaviour.^37^

Our study has found out that 78% of family members are either overweight or obese, which further increase chances of developing diabetes, considering family history of diabetes. Diabetes screening is the crucial strategy for early detection of the disease for better management.^33^ Therefore, there is a need to screen family members for diabetes and comorbidities. Apart from diabetes, overweight and obesity amongst non-diabetic population is associated with hypertension,^38^ low quality of life,^39^ cardiovascular diseases,^40^ breathing problems,^39^ complications of pregnancy^41^ and depression.^41^

Adiposity is related to poor diabetes control and risk of diabetes.^42^ We found that most patients with diabetes and 58% of family members had abdominal obesity. Abdominal obesity amongst patients with diabetes is strongly associated with comorbid conditions, particularly hypertension.^43^ Most patients (75.5%) in our study declared that they have additional disease other than diabetes, and hypertension was the most occurring comorbid condition (89%), which affirms association of adiposity with comorbidity amongst patients with diabetes. Moreover, the presence of comorbid conditions worsens the Quality of Life (QoL).^44^ Nonetheless, this study did not ask for comorbid condition amongst family members. Our study showed that over half of non-diabetic family members had abdominal obesity. It has been found that people with higher WC levels have 5–7 times higher chances of having T2DM.^45^

The benefits of weight loss amongst T2DM had shown to improve glycaemic control, with severe calorie restriction even reversing the progression of T2DM.^46^ Moreover, weight loss amongst patients with diabetes provides benefits that positively impacts treatment and control of metabolic parametres.^47^ Weight loss is also found to be associated with reduced chances of cardiovascular risks amongst patients with diabetes.^48^ A weight loss of about 7% reduces the risk of developing diabetes by 60%.^49,50^ Additional benefits of losing weight include reduced blood pressure, vascular resistance, total blood volume and cardiac output, improvement in insulin resistance and reduction in sympathetic nervous system activity.^48^ World Health Organization,^51^ recommends preventative interventions in diabetes management. Preventative healthcare is cost-effective and cheaper compared with treatment care.^52^ The Department of Health should invest more resources in preventative measures characterised by physical activity and healthy eating^7^ to minimise diabetes complications and new cases, respectively. However, healthcare providers are particularly encouraged to be innovative, passionate and care enough to develop small pilot projects to identify factors contributing to diabetes and obesity in their areas, to devise practical strategies to bring about change. It is fundamentally important for clinics to start by convening a brainstorming session with health team consisting of nurses, dieticians, physiotherapists, social workers, hospital administrators, community leaders and a group patient with diabetes to come up with ideas for pilot projects. The brainstorming session may help in identifying local skills like local chefs who may be interested in developing healthy recipes that are culturally and economically appropriate and recruited to teach cooking classes. The dieticians may be able to identify healthy food options for the chefs to prepare. Equally, physical trainers or bio kinetics can be involved for exercise lessons with the guidance of physiotherapists. The involvement of communities will enable business persons in the area to also take part by funding cooking and exercise classes. Additionally, patients with diabetes with some significant changes in their weight and diabetes through lifestyle changes can be recruited to talk to patients waiting in the diabetic clinic about how they achieved their success.

### Recommendations

There is an urgent need for partnering with family members in the care for patients with diabetes for better control and minimising new cases.

It is recommended to adopt patient-centred and collaborative approach intervention for weight and diabetes management, specifically the 5-A’s methodology be adopted, particularly number two, which is to advise to change with clear, specific, personalised advice.

The study recommends innovation amongst healthcare providers to initiate small pilot projects to identify factors contributing to diabetes and obesity, to devise practical strategies for change.

It is recommended to screen non-diabetic family members for diabetes and any other illnesses considering obesity.

### Limitations of the study

The study did not include the spouses of diabetes patients and also comorbidities amongst family members of patients.

## Conclusion

From this study, we conclude that evidence from various studies suggest that non-diabetic family members of patients with diabetes are at an increased risk of being diagnosed with DM as they already are obese just like patients with diabetes.^21,22^ Therefore, there is an immediate need for primary healthcare facilities to partner with family members in the care for patients with diabetes for better control, reduction of complications and minimising new cases. Patients with diabetes are obese and suffer from comorbidities, which leads to poor quality of life whilst family members are at an increased risk of developing diabetes. As such, it is recommended that family members must be screened for diabetes and comorbidities. It is also important to create conducive environment that discourages sedentary behaviours through lifestyle dietary and exercise modifications to minimise chances of overweight patients and family members progressing to the development of NCDs. Diabetes education should be strengthened and include empowering both patients and their family members with nutrition and exercise management for better outcomes.
